# High-intensity interval training and health-related quality of life in de novo heart transplant recipients – results from a randomized controlled trial

**DOI:** 10.1186/s12955-020-01536-4

**Published:** 2020-08-17

**Authors:** Katrine Rolid, Arne K. Andreassen, Marianne Yardley, Einar Gude, Elisabeth Bjørkelund, Anne R. Authen, Ingelin Grov, Kjell I. Pettersen, Christian H. Dall, Kristjan Karason, Kaspar Broch, Lars Gullestad, Kari Nytrøen

**Affiliations:** 1 Department of Cardiology, Oslo University Hospital Rikshospitalet, Rikshospitalet, , PO Box 4950 Nydalen, N-0424 Oslo, Norway; 2 Institute of Clinical Medicine, Faculty of Medicine, University of Oslo, Oslo, Norway; 3 The Norwegian Health Association, Oslo, Norway; 4 KG Jebsen Center for Cardiac Research, University of Oslo, Norway and Center for Heart Failure Research, Oslo University Hospital, Oslo, Norway; 5 Department of Cardiology, Bispebjerg University Hospital, Copenhagen, Denmark; 6 Transplant Institute, Sahlgrenska University Hospital, Gothenburg, Sweden

**Keywords:** Health-related quality of life, Heart transplantation, High-intensity interval training, Moderate intensity continuous training, Oxygen consumption, Muscle strength,self-reported physical function, Exercise

## Abstract

**Background:**

Studies on the effect of high-intensity interval training (HIT) compared with moderate intensity continuous training (MICT) on health-related quality of life (HRQoL) after heart transplantation (HTx) is scarce. No available studies among de novo HTx recipients exists. This study aimed to investigate the effect of HIT vs. MICT on HRQoL in de novo recipients.

**Methods:**

The HITTS study randomized eighty-one de novo HTx recipients to receive either HIT or MICT (1:1). The HIT intervention were performed with 2–4 interval bouts with an intensity of 85–95% of maximal effort. The MICT group exercised at an intensity of 60–80% of their maximal effort with a duration of 25 min. HRQoL was assessed by the Short Form-36 version 2 (SF-36v2) and the Hospital Anxiety and Depression Scale, mean 11 weeks after surgery and after a nine months’ intervention. The participants recorded their subjective effect of the interventions on their general health and well-being on a numeric visual analogue scale. Clinical examinations and physical tests were performed. Differences between groups were investigated with independent Student t-tests and with Mann-Whitney U tests where appropriate. Within-group differences were analyzed with Paired-Sample t-tests and Wilcoxon Signed Rank tests. Correlations between SF-36 scores and VO_2peak_ were examined with Pearson’s correlations.

**Results:**

Seventy-eight participants completed the intervention. Both exercise modes were associated with improved exercise capacity on the physical function scores of HRQoL. Mental health scores remained unchanged. No differences in the change in HRQoL between the groups occurred except for Role Emotional subscale with a larger increase in the HIT arm. Better self-reported physical function was associated with higher VO_2peak_ and muscle strength.

**Conclusion:**

HIT and MICT resulted in similar mean changes in HRQoL the first year after HTx. Both groups experienced significant improvements in the physical SF-36v2.

**Trial registration:**

ClinicalTrials.gov number: NCT01796379 Registered 18 February 2013.

## Background

Heart transplantation (HTx) is the preferred therapy for selected patients with end-stage heart failure [1]. To improve physical capacity and health-related quality of life (HRQoL), cardiac rehabilitation is an integrated component in most HTx programs.

HRQoL is impaired prior to transplantation [2–4]. Longitudinal studies have reported that HRQoL improves significantly after HTx, with the greatest improvement occurring during the first half year [2, 5, 6]. Most of the studies assessing long-term HRQoL after HTx have shown that HRQoL remains good up to five, [2, 7] ten [8] and up to 20 [9] years after transplant.

The physical domains in HRQoL are lower in HTx recipients than in the general population [1, 10], while the mental health domains has been found comparable to the general population [7, 8]. The physical functioning subscale in the Short-Form-36 (SF-36v2) is related to peak oxygen consumption (VO_2peak_), reflecting an association between self-reported physical function and objective measurements [11, 12]. The impact of exercise capacity on HRQoL has been studied at different time points after HTx [11–21]. Studies have found an association between improved exercise capacity and HRQoL [11, 19], but the effect of different exercise modes on HRQoL is unclear [1], mainly due to lack of high-quality studies. Only one small study has examined the effect of high-intensity interval training (HIT) vs. moderate intensity continuous training (MICT) on HRQoL in maintenance HTx recipients, but found no difference between the two groups [13]. The effect of HIT vs. MICT on HRQoL in newly heart transplanted recipients has not been studied, but these patients may have a greater potential for improvement in HRQoL [1].

The aim of this study was to investigate the effect of HIT vs. MICT the first year after heart transplantation. We hypothesized that HIT would improve HRQoL more than MICT in de novo HTx recipients.

## Methods

**Fig. 1:**
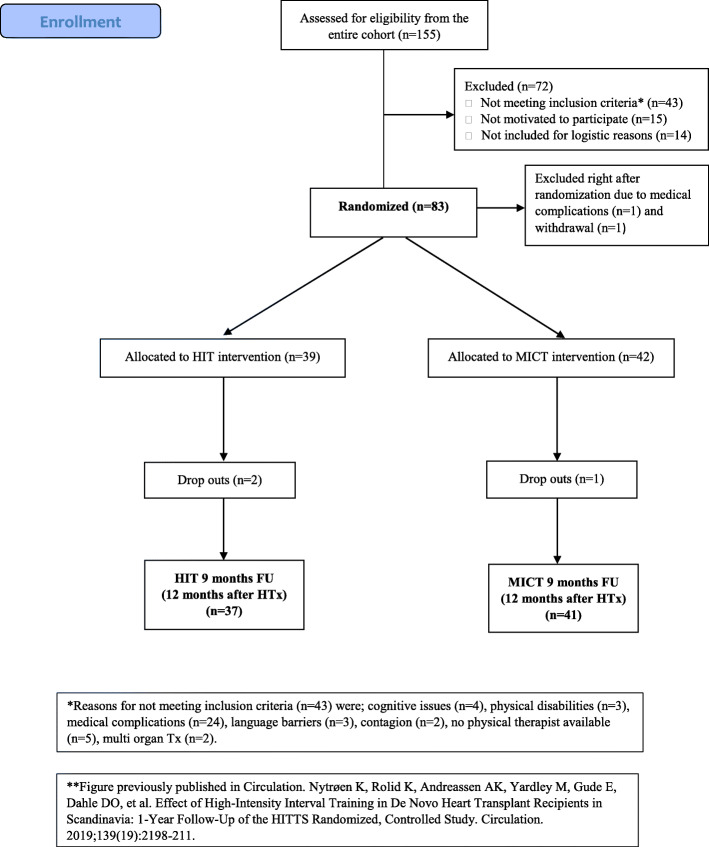
Patient recruitment and follow-up

The study-design and other results has been described earlier [22, 23]. In short, it was a multi-center, randomized controlled trial comparing HIT vs. MICT in adult, consenting de novo HTx recipients. The trial was conducted at three transplant-centers in Scandinavia. The primary endpoint for the overall project was the change in VO_2peak_, while the prespecified endpoint for this substudy was the change in HRQoL. Eighty-one participants were included 7–16 weeks after HTx, and 78 were retested after nine months (Fig. 1). A permuted block randomization list was computer generated by a third party. Numbered sealed envelopes detailing the individual treatment allocation was prepared based on this list. Participants were assigned a randomization number at inclusion. After the CPET test at baseline, the envelope was opened and the patient was allocated to HIT or MICT.

### Exercise intervention

The intervention is described elsewhere [22, 23]. Briefly, the participants were randomized 1:1, to either nine months of HIT or MICT at 11 ± 2 weeks after HTx. Participants in both groups exercised 2–3 times per week in the 9-month long intervention. The HIT consisted of 2–4 interval bouts at an intensity of 85–95% of maximal effort (corresponding to a rated perceived exertion (RPE) of 16–18). Between the HIT bouts, there was an active rest period (RPE 11–13). The goal for the HIT group was to be able to perform 4 interval bouts of 4 min length in the last intervention period. The MICT group followed the standard-of care exercise recommendations in recently HTx recipients, with an exercise intensity of 60–80% of maximal effort (corresponding to an RPE of 12–15) for a duration of 25 min. Both interventions included a 10 min warm up and a cool-down period of 5 min at the end of the exercise session. In addition, both groups performed strength training. All exercise sessions were performed in the participants’ local communities, supervised by health personnel and all exercise sessions in both groups were logged and monitored with a heart rate monitor. Of 72 planned sessions, the HIT group completed median (interquartile range (IR)) 60 (28) sessions and the MICT group completed 56 (37) (p for difference 0.858).

### Self-reported questionnaires

HRQoL was assessed by the generic questionnaire SF-36v2, [24] frequently used in HTx populations [1, 25]. The SF-36 is divided into eight subscales; Physical Functioning, Role Physical, Bodily Pain, General Health, Vitality, Social Functioning, Role Emotional and Mental Health. The eight subscales aggregate into two summary scores; a Physical Component and a Mental Component; higher score indicating better HRQoL. In this study, all scores were transformed to norm-based values with a standardized mean of 50 and a standard deviation (SD) of 10. A change of 2–4 points on any item is considered to be of clinical significance [24].

Symptoms of anxiety and depression were measured with the Hospital Anxiety and Depression Scale (HADS) [26]. The participants’ socio-demographic background was assessed by a simple questionnaire at baseline and at follow-up. Additionally, at follow-up, all the participants recorded: *“To what extent do you feel participation in this study had a positive impact on your general health and well-being”* on a numeric visual analogue scale (VAS), ranging from “not at all” to “to a very great extent”.

**Fig. 2:**
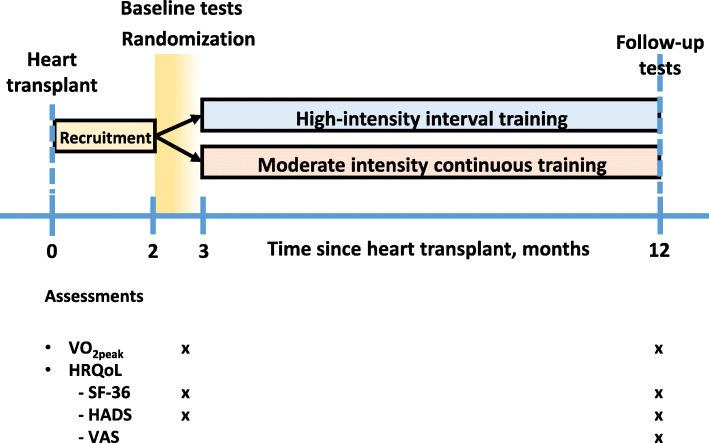
Design of the study

All the questionnaires were self-administered and filled out during the study visits at both time points (Fig. 2). The Physical Functioning subscale from SF-36v2 was selected to represent self-reported physical function.

### Exercise testing

All participants underwent a cardiopulmonary exercise test (CPET) with measurements of VO_2peak_ at baseline and at follow-up. Most of the Norwegian participants (*n* = 70) in were tested on a treadmill with breath-by-breath gas analysis (Jaeger® Masterscreen® CPX, Carefusion), and four of the particpants were tested on a bicycle (Schiller Cardiovit® CS-200 Excellence). The participants from Sweden and Denmark (n = 7) were tested on a bicycle (Jaeger®,Oxy Con Pro® and Jaeger® Vyntus® CPX). The CPET tests was performed with an individualized protocol with a gradual increase in workload until exhaustion [22, 27]. Isokinetic muscle strength and muscular exercise capacity in the lower limbs were measured with a dynamometer (Cybex 6000) [22, 23, 28].

### Ethics

All participants provided written informed consent prior to inclusion. The study was approved by the regional ethic committees in Norway, Sweden and Denmark. The study is conducted according to the Helsinki Declaration. https://clinicaltrials.gov/ identifier NCT01796379.

### Statistics

Continuous data are expressed as mean ± SD, or median (IR). Categorical data are presented as number and percentages. An intention-to-treat analysis were conducted. Differences between the two groups were investigated with independent Student t -tests and with Mann-Whitney U tests where appropriate. The change (delta value) for each participant between baseline and 1-year follow-up was calculated by subtracting the results at 1-year follow-up with the results at baseline [Change = 1-year follow-up – baseline]. The change was assessed by independent t-tests to calculate the mean difference in change between the two groups in normally distributed variables, and by Mann-Whitney U tests for variables with skewed distribution. Within-group differences were analyzed with Paired-Sample t-tests and Wilcoxon Signed Rank tests. We assessed associations between HRQoL scores and parameters reflecting exercise capacity using Pearson’s and Spearman’s correlations. Missing data in the SF-36v2 were handled by the “half-scale” rule, which means that a scale score was calculated if at least half of the items of that specific scale were answered [24]. For the two HADS scales, scores were calculated for those with complete data only. All data were analyzed using IBM SPSS 25.0 (IBM Corporation, United States). *P* values < 0.05 (two-sided) were considered statistically significant.

## Results

Demographic data are provided in Table 1. There were no differences between the intervention arms regarding baseline socio-demographic or clinical characteristics.

**Table 1:** Baseline characteristics in the HIT group and the MICT group^a^

Variables	HIT (*n* = 37)	MICT (*n* = 41)
Sex n (%) men	28 (76)	29 (71)
Age (years)	50 ± 12	48 ± 14
Body Mass Index kg/m^2^	24.8 ± 3.4	25.6 ± 3.9
In a relationship (married/cohabitant)	22 (61)	30 (73)
Employed	8 (22)	9 (22)
Primary diagnosis n (%)		
CM/CAD/Other	21 (57) / 14 (38) / 2 (5)	31 (75) / 6 (15) / 4 (10)
Donor age (years)	37 ± 14	39 ± 14
Ischaemic time (min)	181 ± 77	184 ± 82
Median (IR) years of HF duration pre HTx	4.0 (9.1)	4.5 (8.1)
Median (IR) days on waitlist	85 (192)	71 (167)
Smoking (n (%) No/Ex-smoker)	18 (49) / 19 (51)	21 (51) / 20 (49)
Biomarkers		
Hemoglobin (g/dL)	11.8 ± 1.8	11.7 ± 1.6
Creatinine (μmol/L)	116.1 ± 33.9	118.5 ± 28.0
eGFR (mL/min/1.73m^2^)	65.1 ± 20.9	62.7 ± 23.3
HbA1c (%)	5.7 ± 0.9	5.6 ± 0.7
Medication at inclusion n (%)		
Ciclosporine	24 (65)	31 (76)
Tacrolimus	11 (30)	10 (24)
Everolimus	12 (32)	13 (32)
Prednisolone	37 (100)	41 (100)
Mycophenolate	34 (92)	36 (88)
Statin	36 (97)	41 (100)
Beta blocker	9 (24)	12 (30)
Calcium blocker	8 (22)	12 (30)
ACE inhibitor	0	2 (5)
ARB	4 (11)	3 (8)
Diuretics	31 (84)	32 (78)

Variables are presented as mean ± standard deviation, median (interquartile range (IR)) or number (percentages). *ACE* angiotensin converting enzyme, *ARB* angiotensin II reseptor blocker, *CAD* coronary artery disease, *CM* cardiomyopathy, *eGFR* estimated glomerular filtration rate (Chronic Kidney Disease Epidemiology Collaboration calculation), *HbA1c* hemoglobin A1c, *HF* heart failure, *HIT* High-intensity interval training, *HTx* heart transplantation, *MICT* moderate intensity contionuous training

^a^No difference between groups

All HRQoL variables were similar in the two groups at baseline. Symptoms of depression and anxiety were low in both groups at baseline (Table 2).

**Table 2:** Baseline and follow-up results in the HIT group and the MICT group^d^

Variables	Baseline	Follow-up HIT (*n* = 37)	t-test, *P* value	Baseline	Follow-up MICT (*n* = 41)	t-test, *P* value
Health-related quality of life SF-36v2 components summaries and subscales						
Physical Component Summary (PCS)	42.2 ± 7.6	48.4 ± 9.3	< 0.001	43.2 ± 7.7	49.0 ± 8.4	< 0.001
Mental Component Summary (MCS)	52.5 ± 12.9	53.4 ± 11.9	0.673	55.1 ± 8.2	52.5 ± 9.6	0.086
Physical Functioning	45.0 ± 7.0	50.8 ± 6.0	< 0.001	46.4 ± 6.4	51.6 ± 6.6	< 0.001
Role Physical	37.6 ± 10.4	48.1 ± 9.3	< 0.001	40.8 ± 10.0	47.0 ± 10.0	< 0.001
Bodily Pain	47.8 ± 9.3	50.5 ± 10.5	0.163	48.1 ± 9.2	49.1 ± 12.2	0.583
General Health	48.2 ± 9.4	50.8 ± 11.0	0.067	49.8 ± 7.3	51.2 ± 9.4	0.292
Vitality	50.6 ± 10.8	52.6 ± 12.7	0.196	51.2 ± 9.4	53.6 ± 9.0	0.031
Social Functioning	46.7 ± 9.9	50.2 ± 9.1	0.047	48.7 ± 8.7	50.7 ± 9.0	0.278
Role Emotional	46.8 ± 13.1	52.0 ± 9.1	0.027	50.7 ± 7.7	48.7 ± 10.5	0.246
Mental Health	53.1 ± 11.0	53.7 ± 9.7	0.684	55.4 ± 7.8	54.0 ± 9.7	0.232
HADS Anxiety median (IR)	3.0 (3.5)	2.0 (4.5)	0.310^a^	3.0 (3.5)	3.0 (4)	0.400^a^
HADS Depression median (IR)	2.0 (3.5)	2.0 (4.8)	0.331^a^	1.0 (2.5)	1.0 (3.8)	0.866^a^
VAS scale (0–100) median (IR)^b^		82.0 (20.5)			75.5 (37.3)	0.235^c^
Cardiopulmonary exercise test						
VO_2peak_ (mL/kg/min)	19.5 ± 4.3	24.4 ± 6.5	< 0.001	21.3 ± 5.3	24.4 ± 6.7	< 0.001
% of predicted VO_2peak_	53.3 ± 11.6	66.6 ± 15.4	< 0.001	58.4 ± 12.5	66.9 ± 14.8	< 0.001
RPE (Borg scale score)	18.7 ± 0.5	18.8 ± 0.6	0.290	18.5 ± 1.1	18.8 ± 0.7	0.098
RER	1.17 ± 0.11	1.19 ± 0.1	0.314	1.22 ± 0.13	1.22 ± 0.1	0.751
Muscular capacity						
Maximal muscle strength extensors (Newton meter)	184 ± 74	237 ± 81	< 0.001	186 ± 73	222 ± 80	< 0.001
Muscular exercise capacity extensors (Joule)	2154 ± 952	3170 ± 1267	< 0.001	2319 ± 1201	2870 ± 1240	< 0.001

Health-related quality of life, exercise capacity and muscular strength at baseline (~ 11 weeks after HTx and at 9 months intervention (first yearly annual follow-up). Variables are presented as mean ± standard deviation or median (Interquartile range (IR)).*HADS* Hospital Anxiety and Depression Scale, *HIT* High-intensity interval training, *MICT* moderate intensity contionuous training, *RER* Respiratory exchange ratio, *RPE* Rated perceived exertion, *VAS* visual analogue scale

^a^Wilcoxon Signed Rank Test

^b^Measured at follow-up only

^c^Mann-Whitney U test (difference between groups at follow-up)

^d^No difference between groups at baseline

During the intervention, the scores for the SF-36v2 subscales Physical Functioning and Role Physical improved significantly in both exercise arms (Table 2). The improvement in these scales exceeded two points, which is regarded a clinically important difference [24]. Accordingly, the Physical Component Summary scores improved significantly (Table 2). The Mental Component Summary scores were above 50 at baseline, while HADS scores were low. Neither the Mental Component Summary scores nor the HADS scores did change significantly during the intervention period (Table 2).

The participants’ general health and well-being was good, as shown on the VAS scale. At follow-up, the HIT group scored 82 points and the MICT group scored 76 (p for difference = 0.235) (Table 2).

**Table 3:** Comparison of change between the HIT group and the MICT group from baseline to follow-up

Variables	Change within the HIT group Mean ± SD (*n* = 37)	Change within the MICT group Mean ± SD (*n* = 41)	Difference in mean change between groups mean [95% CI]	*P* value Difference in change between groups
Health-related quality of life SF-36v2 components summaries and subscales				
Physical Component Summary (PCS)	6.3 ± 8.2^**^	5.7 ± 5.7^**^	0.6 [− 3.1, 4.2]	0.762
Mental Component Summary (MCS)	0.9 ± 12.5	− 2.6 ± 9.3	3.4 [− 1.5, 8.5]	0.170
Physical Functioning	5.8 ± 5.6^**^	5.2 ± 5.6^**^	0.6 [− 2.0, 3.2]	0.653
Role Physical	10.5 ± 11.2^**^	6.2 ± 10.0^**^	4.3 [− 0.6, 9.1]	0.082
Bodily Pain	2.7 ± 11.5	1.0 ± 11.4	1.7 [− 3.5, 6.9]	0.509
General Health	2.6 ± 8.3	1.4 ± 8.6	1.1 [− 2.7, 4.9]	0.555
Vitality	2.0 ± 9.2	2.6 ± 7.3^*^	− 0.6 [− 4.3, 3.2]	0.760
Social Functioning	3.6 ± 10.5^*^	2.0 ± 11.6	1.5 [− 3.5, 6.5]	0.541
Role Emotional	5.2 ± 13.4^*^	− 2.0 ± 11	7.2 [1.6, 12.8]	0.012
Mental Health	0.6 ± 9.0	− 1.4 ± 7.3	2.0 [− 1.7, 5.7]	0.284
HADS Anxiety	−1.0^a^	−0.8^a^		0.920^c^
HADS Depression	−1.0^b^	−0.2^a^		0.427^c^
Cardiopulmonary exercise test				
VO_2peak_ (mL/kg/min)	4.8 ± 4.1^**^	3.1 ± 3.5^**^	1.8 [0.1, 3.5]	0.044
Improvement in mL/kg/min (%)	25.2 ± 21.1^**^	15.1 ± 17.8^**^	10.1 [1.3, 19.0]	0.025
% of predicted VO_2peak_	13.2 ± 10.7^**^	8.5 ± 9.1^**^	4.7 [0.2, 9.2]	0.040
RPE (Borg scale score)	0.1 ± 0.8	0.3 ± 1.2	0.2 [−0.3, 0.7]	0.424
RER	0.02 ± 0.1	−0.01 ± 0.1	0.02 [− 0.03, 0.1]	0.338
Muscular capacity				
Maximal muscle strength extensors (Newton meter)	54 ± 49^**^	36 ± 34^**^	178 [− 3, 39]	0.094
Muscular exercise capacity extensors (Joule)	1016 ± 812^**^	551 ± 780^**^	464 [63, 863]	0.024

Health-related quality of life, exercise capacity and muscular strength at baseline (~ 11 weeks after HTx and at 9 months intervention (first yearly annual follow-up). Variables are presented as mean ± standard deviation. *CI* Confidence Interval, *HADS* Hospital Anxiety and Depression Scale, *HIT* High-intensity interval training, *MICT* moderate intensity contionuous training, *SD* standard deviation, *RER* Respiratory exchange ratio, *RPE* Rated perceived exertion, *VAS* visual analogue scale

Within group: ^**^p < 0.001, ^*^p < 0.05

^a^Based on negative ranks

^b^Based on positive ranks

^c^Mann-Whitney U test

As reported earlier, there was a significant between-group difference in increased VO_2peak_ over the intervention period, in favor of HIT [23] (Table 3). However, there were no differences between the two exercise arms in HRQoL, the main endpoint of this substudy, except on the Role Emotional subscale, which covers the spectrum of mental health-related role constraints related to work or other daily activities [24] (Table 3). Maximal RPE (Borgs scale score) were equal between the two groups and did not change during the intervention period [23] (Table 3).

There were no differences between groups regarding rejections or serious/adverse events during the intervention period [23].

**Fig. 3:**
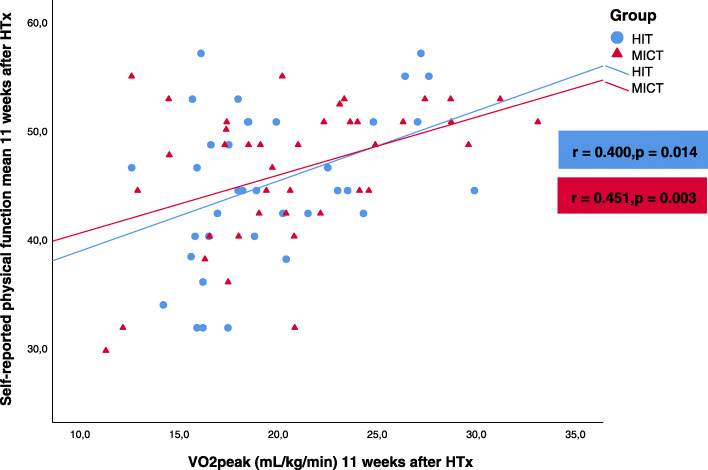
Correlation between self-reported physical function and VO_2peak_ in the high-intensity training (HIT) group and the moderate intensity continuous training (MICT) group mean 11 weeks after heart transplantation (HTx)

**Fig. 4:**
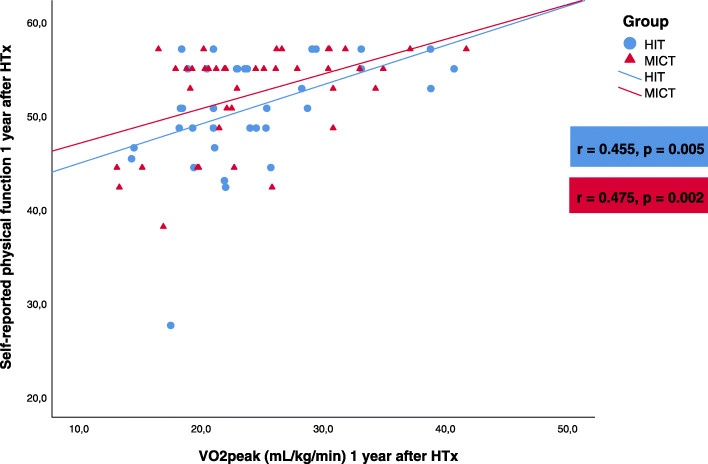
Correlation between self-reported physical function and VO_2peak_ in the high-intensity training (HIT) group and the moderate intensity continuous training (MICT) group 1 year after heart transplantation (HTx)

There was a positive correlation between VO_2peak_ and the self-reported physical function in both groups, both at baseline (Fig. 3) and at follow-up (Fig. 4). In the HIT group, we found a modest correlation between the change from baseline to 1-year follow-up in self-reported physical function and the change in VO_2peak_ (Pearson’s *r* = 0.35, *p* = 0.03). There was no correlation between the corresponding changes in the MICT group (Pearson’s *r* = − 0.13, *p* = 0.41).

The self-reported physical function also correlated with the extensors’ maximal muscle strength and muscle endurance at both time points in both groups (See Additional File 1, Figs. 1, 2, 3 and 4).

The SF-36 Role Physical scale correlated modestly with VO_2peak_ in both groups at 1-year follow-up. Correlations between other CPET values (heart rate variables, O_2_ pulse, maximal ventilation, respiratory exchange ratio, RPE) and SF-36 subscales were weak in both groups at both time points. However, there was a moderate correlation between metabolic equivalents and self-reported physical function in both groups at both time points (data not shown).

### Missing data in the questionnaires

There was little missing data. At baseline there were 1.3% missing for the following SF-36 subscales; Role Physical, Vitality and Mental Health and 2.6% missing for the Role Emotional subscale and each of the two SF-36 sum scores. At follow-up there were 1.3% missing for all of the SF-36 subscales except of General Health and Social Functioning, while there were 2.6% missing for each of the two SF-36 sum scores and for each of the HADS scores.

## Discussion

The main findings in the present study were: 1) In patients who had recently undergone HTx, the Physical Component Scores improved significantly during the nine-months long intervention period, and 2) There were no differences in HRQoL between patients allocated to HIT or MICT, except on the Role Emotional subscale where the HIT group had a significantly higher score.

Maintenance HTx recipients tend to score lower than the general population on the physical function domains of HRQoL [7, 8]. Interventions to improve physical function in HTx recipients are of special interest since improved physical function is associated with better HRQoL [11, 17] and is a strong predictor for survival [12].

In exercise trials comparing HIT with a control group in maintenance HTx recipients, improvements in general health is higher in the intervention groups [14, 15]. These results suggest that exercise has a positive effect on HRQoL in the long term after HTx. In line with our findings, Hsu et al. [16] observed improved HRQoL in the physical function domains of SF-36 after cardiac rehabilitation early after HTx. It should be noticed that neither our study, nor the study by Hsu et al., [16] had a control group without an exercise program. The relatively high HRQoL observed at the end of our trial, and in the study by Hsu et al. [16] may reflect an overall improved health status during the first year after HTx, rather than an effect of exercise alone. For example, Ortega et al. [29] found improvements in SF-36 physical domains over the first year after HTx without an intervention.

To our knowledge, only one prior study has investigated the effect of HIT vs. MICT on HRQoL in HTx recipients [13]. In this crossover trial (*n* = 16), [13] there were no differences between the groups regarding HRQoL, symptoms of anxiety or depression, which is in line with our results. However, the same study found a significant decrease in symptoms of anxiety in the HIT group, and a significant decrease in symptoms of depression in both groups. This contrasts our study, where symptoms of depression and anxiety were low and stable throughout the intervention period in both groups.

The improvement in the Role Emotional subscale in our patients randomized to HIT may reflect an improved sense of achievement associated with exhaustive exercise, but may also be an incidental finding.

We found correlations between VO_2peak_ and self-reported physical function at both time points, as previously reported in maintenance HTx recipients [19]. The correlation between the change in self-reported physical function and the change in VO_2peak_ from baseline to 1-year follow-up was observed in the HIT group only. This may be due to the higher mean change in VO_2peak_ in the HIT group compared to the MICT group. VO_2peak_ and self-reported physical function are strong predictors for long-term survival after HTx [12]. Obtaining self-reported physical function is less resource-demanding than performing CPET with measurements of VO_2peak_. However, the correlation between the two is modest, and self-reported physical function cannot fully substitute VO_2_ measurements in the short and in the longer term after HTx.

### Limitations

The high baseline HRQoL scores may reflect an above average healthy population and may also have affected the impact of the intervention on HRQoL. For obvious reasons, the sickest patients could not be enrolled in the trial. Thus, our results may not be valid for the entire HTx population. HRQoL was a secondary, but prespecified endpoint in the HITTS (High-intensity Interval Training in De Novo Heart Transplant Recipients in Scandinavia) study [22, 23]. With only 78 participants we may face a type II error due to insufficient statistical power.

A disease-specific HRQoL questionnaire could have been more sensitive to detect differences between groups. So far, no disease-specific questionnaires in Norwegian are available for the HTx population. In a prior HTx study, we experienced a ceiling effect using the heart failure-specific Kansas City Cardiomyopathy Questionnaire [30] and decided not to use this questionnaire in this study.

The HITTS trial [22, 23] was not designed to assess the participants´ daily activities and the roles they were hoping to assume*.* This limits our ability to explain the between-group difference in the Role Emotional scale.

### Clinical implications and future directions

Interventions for good and stable HRQoL, both short- and long-term after HTx, are needed. Exercise yields better physical function and makes it easier to engage in various activities of everyday life. However, despite improved VO_2peak_ with the HIT intervention, HRQoL was similar in both intervention arms. The development of an organ transplant-specific HRQoL questionnaire is warranted for future research in this field, [25] as it probably will be more accurate to detect changes in health status associated with organ transplant issues.

## Conclusion

This randomized controlled trial demonstrated significant improvements in the physical function components in HRQoL over a nine-month long exercise intervention in de novo HTx recipients. However, despite a larger improvement in exercise capacity in the HIT group, there were no between-group differences regarding the change in HRQoL.

## Supplementary information

**Additional file 1: Figure 1.** Correlation between self-reported physical function and maximal muscle strength in the high-intensity training group and the moderate intensity continuous training group 11 weeks after heart transplantation (HTx). **Figure 2.** Correlation between self-reported physical function and maximal muscle strength in the high-intensity training group and the moderate intensity continuous training group 1 year after heart transplantation (HTx). **Figure 3.** Correlation between self-reported physical function and muscle endurance in the high-intensity training group and the moderate intensity continuous training group 11 weeks after heart transplantation (HTx). **Figure 4.** Correlation between self-reported physical function and muscle endurance in the high-intensity training group and the moderate intensity continuous training group 1 year after heart transplantation (HTx).

## Data Availability

The data generated and analyzed during the current study are not publicy available due to Norways strict guidelines for privacy policy and data sharing.

## Abbreviations

CPET: Cardiopulmonary exercise test
HADS: Hospital Anxiety and Depression Scale
HIT: High-intensity interval training
HITTS: High-intensity Interval Training in De Novo Heart Transplant Recipients in Scandinavia
HRQoL: Health-related quality of life
HTx: Heart transplantation/Heart transplant
IR: Interquartile range
MICT: Moderate intensity continuous training
RPE: Rated perceived exertion
SD: Standard deviation
SF-36v2: Short Form-36 version 2
VAS: Visual analogue scale
VO_2peak_: Peak oxygen consumption
