# Supplementary material for: High-intensity interval training and health-related quality of life in de novo heart transplant recipients – results from a randomized controlled trial
Source: Health Qual Life Outcomes. 2020 Aug 17;18:283. doi: 10.1186/s12955-020-01536-4 (PMC7433122; doi:10.1186/s12955-020-01536-4)

**Additional file 1**

Figures showing correlations between self-reported physical function and muscle strength in the high-intensity interval training group and the moderate intensity continuous training group at 11 weeks and 1 year after heart transplantation.

**Additional Figure 1.** Correlation between self-reported physical function and maximal muscle strength in the high-intensity training group and the moderate intensity continuous training group 11 weeks after heart transplantation (HTx).

**Additional Figure 2**. Correlation between self-reported physical function and maximal muscle strength in the high-intensity training group and the moderate intensity continuous training group 1 year after heart transplantation (HTx).

**Additional Figure 3.** Correlation between self-reported physical function and muscle endurance in the high-intensity training group and the moderate intensity continuous training group 11 weeks after heart transplantation (HTx).

**Additional Figure 4** Correlation between self-reported physical function and muscle endurance in the high-intensity training group and the moderate intensity continuous training group 1 year after heart transplantation (HTx) .

**Additional Figure 1**


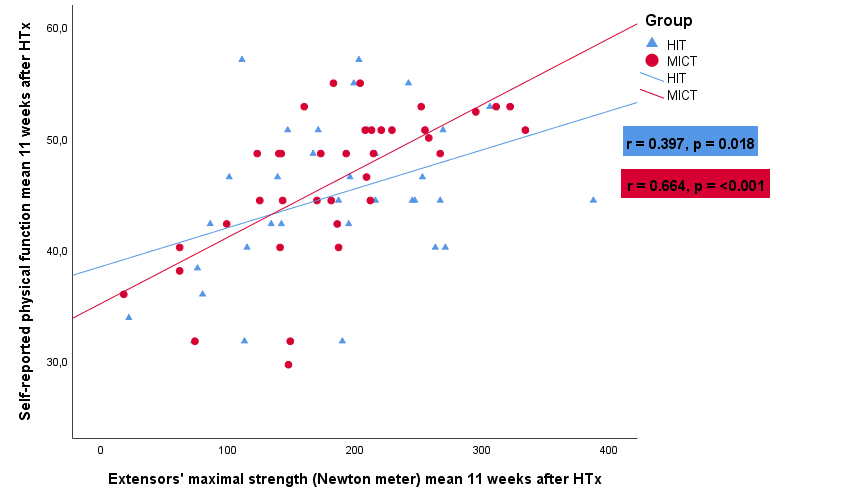


**Additional Figure 2**


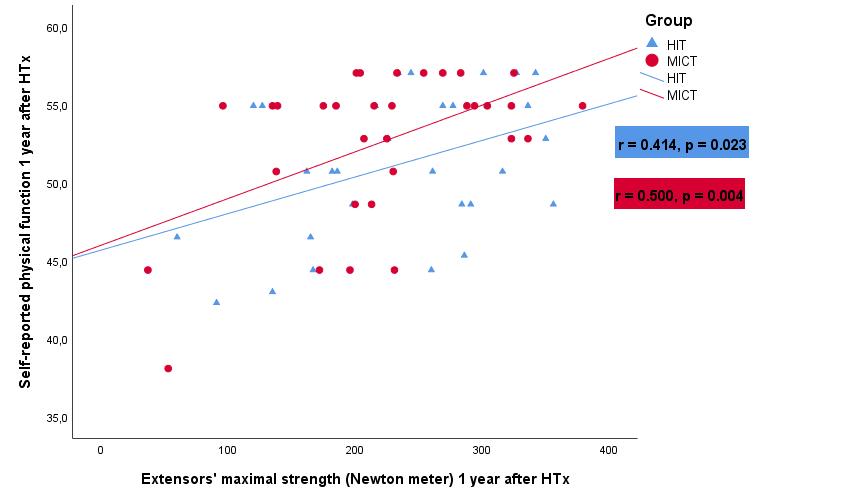


**Additional Figure 3**


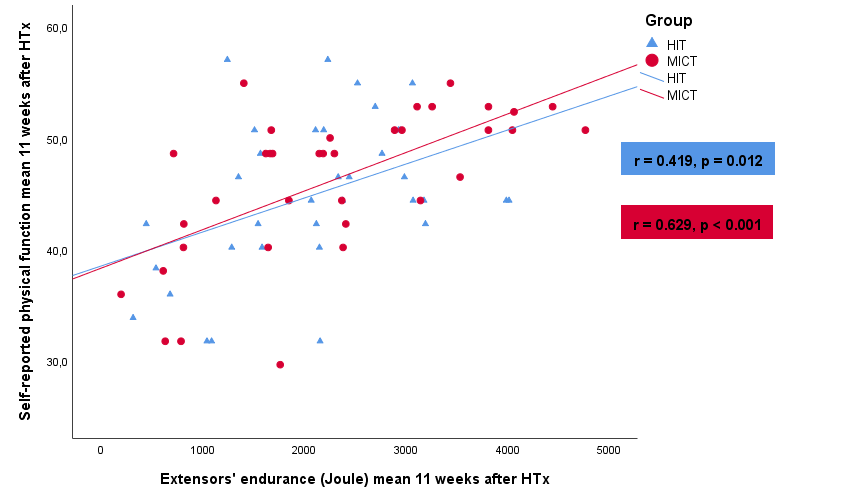


**Additional Figure 4**


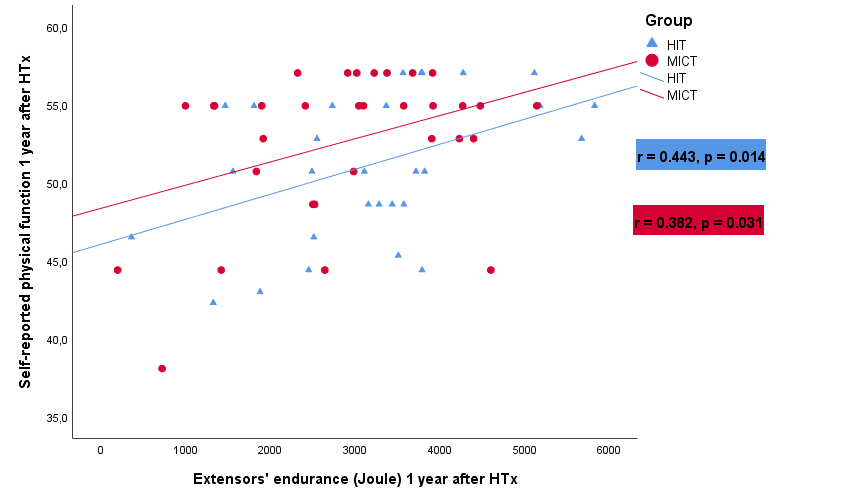

Supplement: Supplementary file 1 — Additional file 1: Figure 1. Correlation between self-reported physical function and maximal muscle strength in the high-intensity training group and the moderate intensity continuous training group 11 weeks after heart transplantation (HTx). Figure 2. Correlation between self-reported physical function and maximal muscle strength in the high-intensity training group and the moderate intensity continuous training group 1 year after heart transplantation (HTx). Figure 3. Correlation between self-reported physical function and muscle endurance in the high-intensity training group and the moderate intensity continuous training group 11 weeks after heart transplantation (HTx). Figure 4. Correlation between self-reported physical function and muscle endurance in the high-intensity training group and the moderate intensity continuous training group 1 year after heart transplantation (HTx). [file 12955_2020_1536_MOESM1_ESM.docx]
